# Effect of Chemotherapy, Laparoscopy, and Cytology on Stage IC Ovarian Clear Cell Carcinoma: A Long-Term, Single-Center Study

**DOI:** 10.3390/ijerph17020491

**Published:** 2020-01-13

**Authors:** Hao-Ting Chang, Mei-Ling Chiu, Tao-Yuean Wang, Tzu-Chien Chen, Chih-Long Chang, Tsung-Hsien Su, Kuo-Gong Wang, Kung-Liahng Wang, Yuh-Cheng Yang, Jen-Ruei Chen

**Affiliations:** 1 Department of Obstetric & Gynecology, MacKay Memorial Hospital, Taipei 10449, Taiwan; haoting79@hotmail.com (H.-T.C.); chen.4418@mmh.org.tw (T.-C.C.); ccl@mmh.org.tw (C.-L.C.); aikuo7@mmh.org.tw (K.-G.W.); eugene@ms2.mmh.org.tw (Y.-C.Y.); 2 Department of Pathology, MacKay Memorial Hospital, New Taipei City 25160, Taiwan; dorchiou@mmh.org.tw (M.-L.C.); tywang@mmh.org.tw (T.-Y.W.); 3 Department of Obstetric & Gynecology, MacKay Memorial Hospital, Hsinchu Branch, Hsinchu 30071, Taiwan; sutsung@ms2.mmh.org.tw; 4 Department of Obstetric & Gynecology, MacKay Memorial Hospital, Taitung Branch, Taitung 95054, Taiwan; klwang@mmh.org.tw; 5 MacKay Junior College of Medicine, Nursing and Management, Taipei 11260, Taiwan

**Keywords:** chemotherapy, clear cell carcinoma, cytology, laparoscopy, ovarian cancer, paclitaxel

## Abstract

Ovarian clear cell carcinoma (OCCC) is the second common histology of epithelial ovarian cancer in Taiwan. Stage IC is common, especially during minimally invasive surgery. Adjuvant chemotherapy in stage IC OCCC is unavoidable, and paclitaxel-based chemotherapy in Taiwan is self-paid. However, surgical spillage from minimally invasive surgery as a cause of unfavorable prognosis is still uncertain. The information of patients with stage IC OCCC, corresponding to a period of January 1995 to December 2016, was retrospectively collected following a chart and pathology review. Data regarding surgical methods, cytology status, regimens of adjuvant chemotherapy, survivorship, progression-free survival (PFS), and overall survival (OS) period were analyzed. In total, 88 patients were analyzed, and 64 and 24 patients were treated with paclitaxel- and nonpaclitaxel-based chemotherapy, respectively. Recurrence was identical between the two groups: PFS (47.5 ± 41.36 versus 54.0 ± 53.9 months, *p* = 0.157) and OS (53.5 ± 38.14 versus 79.0 ± 49.42 months, *p* = 0.070). Of the 88 patients, 12 had undergone laparoscopy for histological confirmation before complete open staging surgery; however, their PFS (49.5 ± 46.84 versus 49.0 ± 35.55 months, *p* = 0.719) and OS (56.5 ± 43.4 versus 51.0 ± 32.77 months, *p* = 0.600) were still comparable. Cytology results were only available for 51 patients, and positive washing cytology results seemed to worsen PFS (*p* = 0.026) but not OS (*p* = 0.446). In conclusion, adjuvant nonpaclitaxel chemotherapy and laparoscopic tumor spillage before the staging operation did not worsen the outcome in stage IC OCCC. Positive washing cytology has a negative effect on PFS but not on OS.

## 1. Introduction

Ovarian cancer is the leading cause of death in gynecologic malignancy worldwide [1]. In Taiwan, the incidence and mortality rates of ovarian cancer are steadily increasing. The most common histology of ovarian cancer in Taiwan is serous carcinoma, and the second most common is clear cell carcinoma [2], unlike in the United States, where clear cell carcinoma ranks fourth [3]. Ovarian clear cell carcinoma (OCCC) has a distinct etiology and is frequently associated with endometriosis and ovarian endometrioma [4].

OCCC is a high-grade ovarian cancer with a poor prognostic histology, even in the early stage [5]. Currently, practical guidelines of the National Comprehensive Cancer Network (NCCN) [6] and the European Society of Medical Oncology (ESMO) [7] recommend adjuvant platinum-based chemotherapy following debulking or staging surgery in women with any stage of OCCC to prevent a high incidence of tumor relapse.

Based on numerous clinical trials, paclitaxel (175 mg/m^2^) plus carboplatin (area under curve; AUC 5–6) are the first choices in chemotherapy for epithelial ovarian cancer [6,7]. However, according to the rules of Taiwan’s National Health Insurance, paclitaxel is prescribed for patients with advanced-stage epithelial ovarian cancer (International Federation of Gynecology and Obstetrics (FIGO) stage III–IV. Patients with FIGO stage I–II epithelial ovarian cancer, including OCCC, could only use nonpaclitaxel platinum-based combination chemotherapy (for example, cisplatin (75 mg/m^2^) plus cyclophosphamide (750 mg/m^2^) or carboplatin (AUC5) plus cyclophosphamide (600 mg/m^2^) under insurance coverage. In early-stage patients, even in those with a high-risk histology like OCCC, paclitaxel is self-paid under the current insurance rules [8].

This study investigated if the benefit of using paclitaxel in platinum-based chemotherapy is higher than that of using platinum-based chemotherapy without paclitaxel in patients with FIGO stage IC1–3 (cancer limited in ovary, surgical cancer spillage (IC1), cancer outgrowth at ovarian capsule or fallopian tubes (IC2), or positive cytology of ascites (IC3)) OCCC following complete surgical staging.

In addition, incidental finding of OCCC during minimally invasive surgery is not rare because it is frequently diagnosed initially as endometriosis or endometrioma. Moreover, the survival impact of early-stage OCCC on incidental diagnosis during minimally invasive surgery was also determined.

## 2. Materials and Methods

### 2.1. Data Collection

Medical records were reviewed following approval from the Institutional Review Board of MacKay Memorial Hospital, Taipei, Taiwan (approval number: 20MMHIS002e), and all personal identifiers were anonymized prior to the analysis. All patients had been treated primarily at our institute during 1995–2016, and all OCCC pathologies were reconfirmed by one of the authors, a specialist in gynecological pathology (TY Wang). Pathological slides of patients who underwent primary surgery at other institutes were obtained for histological reconfirmation. All cases were staged using the new staging system after 2014 from FIGO. We excluded patients with mixed-type histology and whose pathological slides were unavailable. Patients with incomplete primary staging, incomplete courses of chemotherapy after primary surgical staging, or insufficient follow-up were also excluded. Basic characteristics are presented in Table 1.

### 2.2. Statistical Analysis

SPSS version 21.0 (IBM, Armonk, New York, NY, USA) was used for all statistical analyses. Comparisons between continuous variables were analyzed using Student’s *t*-test or Wilcoxon–Mann–Whitney test according to the data distribution. Chi-square tests were used to evaluate categorical variables. A multivariate binary logistic regression was performed to identify clinical variables independently associated with chemotherapy administration.

Progression-free survival (PFS) was defined as the time from primary surgery (pathology confirmation) to the date of disease progression or recurrence, ascertained by imaging according to the response evaluation criteria in solid tumors or alive at the last follow-up with no recurrence. Overall survival (OS) was defined as the time from primary surgery (pathology confirmation) to the date of death or alive at the last follow-up. OS and PFS probabilities were obtained using the Kaplan–Meier method, and the log rank test was used to compare the survival curves. A *p* < 0.05 was considered statistically significant.

## 3. Results

### 3.1. Study Population

A total of 88 patients were enrolled in this retrospective study. Basic characteristics are summarized in Table 1. Based on the chemotherapeutic regimens (paclitaxel-based versus nonpaclitaxel-based chemotherapy, detailed regimens are listed at the footnote of Table 1) and surgical approaches (exploratory laparotomic staging procedure, including total hysterectomy, bilateral salpingo-oophorectomy, bilateral pelvic, and para-aortic lymph node dissection, partial omentectomy, and random peritoneal biopsies, versus laparoscopy then laparotomic conversion and complete staging), the patients were divided into four groups.

### 3.2. Comparison of Different Chemotherapies

**Figure 1:**
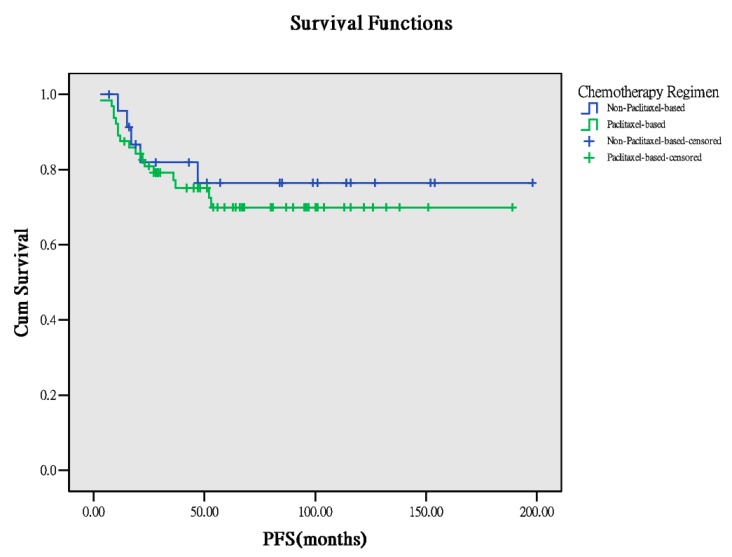
PFS between the different chemotherapeutic regimens (*p* = 0.597).

**Figure 2:**
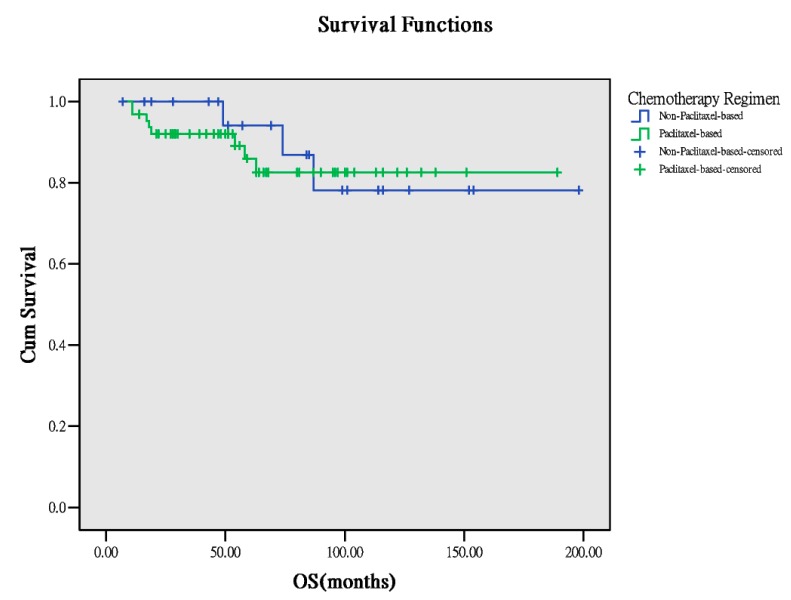
OS between the different chemotherapeutic regimens (*p* = 0.773).

Sixty-four patients received paclitaxel-based chemotherapy, whereas 24 received nonpaclitaxel-based chemotherapy. Basic characteristics (age, body mass index (BMI) at surgery, number of lymph nodes retrieved from surgery), PFS, and OS between both the groups were comparable (Table 1). Notably, PFS and OS demonstrated a worse trend in the paclitaxel-based group than in the nonpaclitaxel-based group (PFS: 47.5 months versus 54.0 months, *p* = 0.157, OS: 53 months versus 79.0 months, *p* = 0.070, Figure 1 and Figure 2). However, the trend was not significant. In the survivorship analyses (Table 2), the two groups still demonstrated comparable results. Thus, paclitaxel-based chemotherapy has a limited advantage in improving outcomes of these patients with early-stage OCCC.

### 3.3. Comparison of Different Surgical Approaches

**Figure 3:**
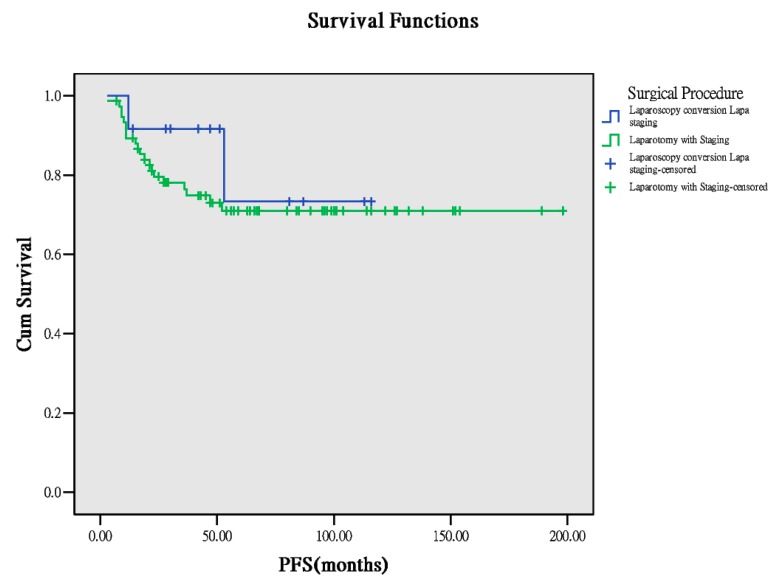
PFS between the surgical approaches (*p* = 0.493).

**Figure 4:**
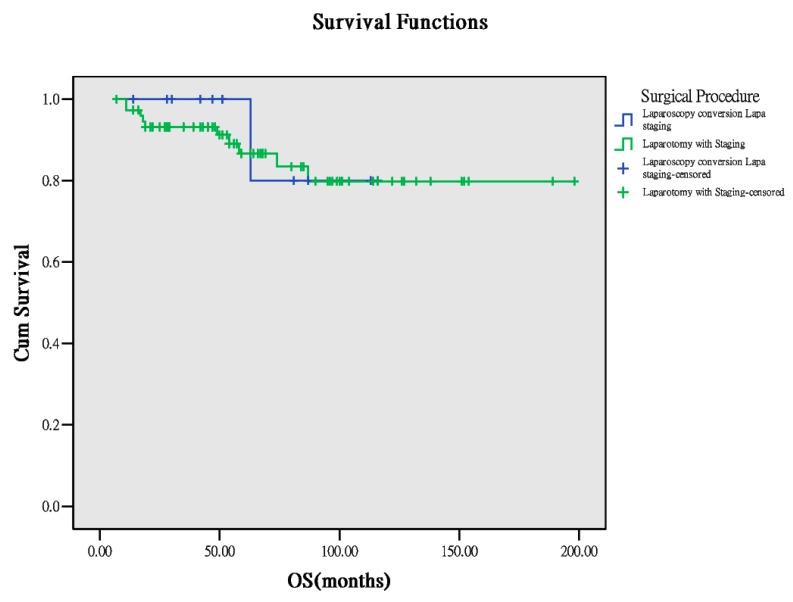
OS between the surgical approaches (*p* = 0.709).

Seventy-six patients received direct exploratory laparotomic staging and 12 patients received laparoscopy, and this was followed by laparotomic staging conversion after confirmation of OCCC diagnosis based on frozen section results. Similarly, basic characteristics (age, body mass index (BMI) at surgery, number of lymph nodes retrieved from surgery), PFS, and OS between both the groups were comparable (Table 1). According to the survivorship analysis, the two groups demonstrated comparable results (Table 3). Survival curves suggested no statistical difference (Figure 3 and Figure 4). This suggests that laparoscopic diagnosis followed by laparotomic conversion for complete staging does not worsen the outcomes in these patients.

### 3.4. Comparison of Cytology Results

**Figure 5:**
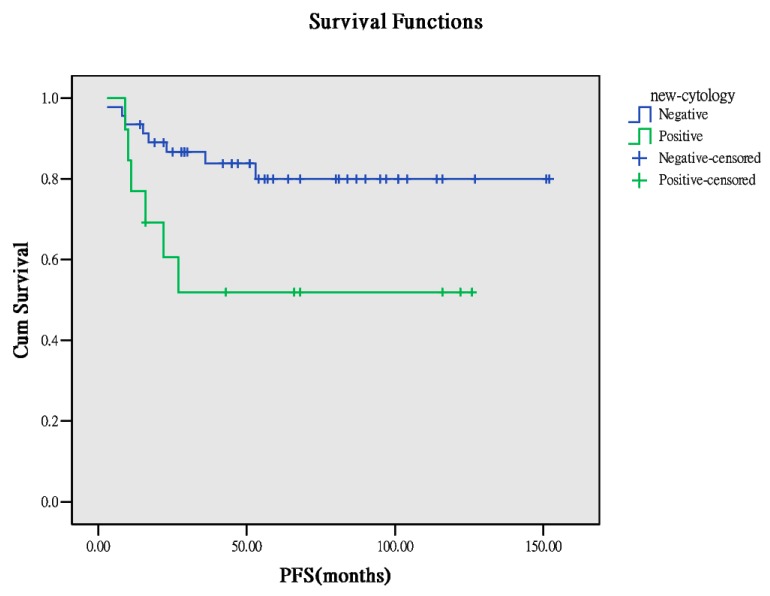
PFS between the cytology results (*p* = 0.026).

**Figure 6:**
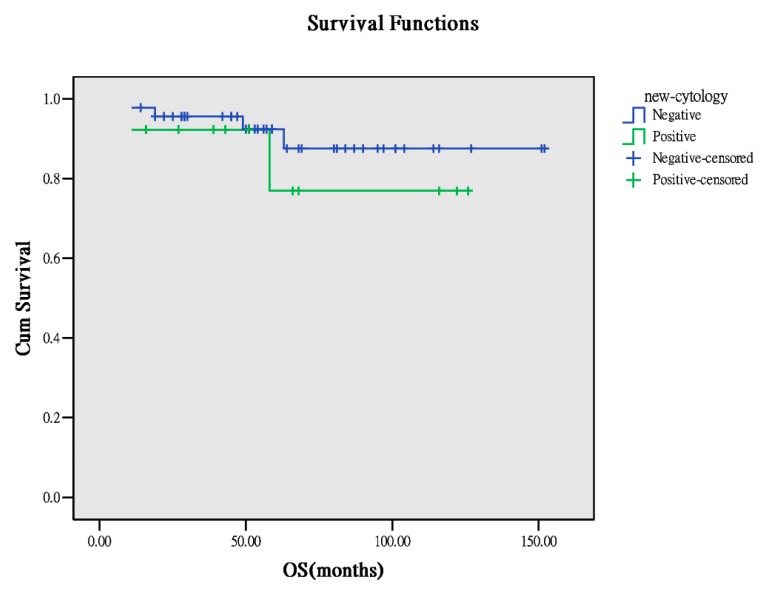
OS between the cytology results (*p* = 0.446).

Furthermore, washing cytology affects the outcome in patients with early OCCC. In our study, the cytology data for analysis were only available for 51 patients. For 38 patients, the peritoneal washing cytology result was negative, whereas for 13 patients, the result was positive. According to the results of recurrence and survivorship analyses for the two groups, a higher trend of recurrence was observed in the positive group (*p* = 0.068), but comparable results were obtained for survivorship analysis (Table 4). Survival curves demonstrated higher PFS in the negative group (*p* = 0.026, Figure 5) than in the positive group. However, no difference in OS was observed (Figure 6). This means that positive washing cytology can lead to high recurrence and poor PFS and has less effect on OS and survivorship.

## 4. Discussion

Ovarian carcinoma is the leading cause of death in gynecologic malignancies worldwide and in Taiwan. Unlike for Western countries, OCCC is the second most common histological type of epithelial ovarian cancer [2], and its pathogenesis is highly correlated to endometriosis [4]. OCCC is classified as high-grade carcinoma, and it has a 33.3% recurrence rate even in early-stage IC, based on a multi-institute retrospective trial in Japan [9]. According to this study, fertility-sparing surgery with contralateral ovary and uterus preservation apparently increases recurrence. Adjuvant chemotherapy with 3–6 courses of a platinum-based regimen was recommended by the current treatment guideline from NCCN [6] and ESMO [7].

Currently, the standard platinum-based chemotherapeutic choice of epithelial ovarian cancer after primary surgical debulking or staging is paclitaxel and carboplatin, based on many clinical trials, especially the Gynecological Oncology Group trial 158 in the United States [10]. However, most enrolled patients had advanced-stage ovarian cancer in this clinical trial, and the advantage of paclitaxel for an early-stage, high recurrence rate subtype of epithelial ovarian cancer is unknown. According to the regulations of Taiwanese National Health Insurance, paclitaxel is administered only to patients with advanced, stage III–IV epithelial ovarian cancer following surgery. In stages I–II, paclitaxel is not covered by the insurance [8], and some patients with OCCC had opted for other alkylating agents (such as cyclophosphamide or ifosfamide) instead of paclitaxel, due to economic limitations. Given the limited clinical data or trials, replacement of paclitaxel with other alkylating agents in stage IC OCCC is controversial.

In our retrospective study, nonpaclitaxel-based chemotherapy showed better trends for PFS and OS than paclitaxel-based chemotherapy in stage IC OCCC, although statistically nonsignificant. This may have been due to the lower number of patients in the nonpaclitaxel group. The same result was observed for survivorship. Our results demonstrated that patients with early-stage OCCC could safely choose nonpaclitaxel agents, such as cyclophosphamide or ifosfamide, for preventing disease recurrence when paclitaxel is unavailable.

OCCC has a significant correlation with pelvic endometriosis and endometrioma according to many cohort studies [4]. Laparoscopy is the gold standard for endometriosis diagnosis. Many cases with early-stage OCCC have similar presentation as ovarian endometrioma or endometriosis and laparoscopy would be performed. OCCC is sometimes found incidentally during enucleation of an ovarian cyst, and tumor rupture during surgical tumor enucleation is frequent. Theoretically, laparoscopic surgical spillage of OCCC increases the risk of peritoneal dissemination and port-site metastases and worsens prognosis [11]. In this study, we identified patients who first received laparoscopic surgery and then converted to laparotomic surgical staging after confirming OCCC diagnosis through frozen sectioning during operation. Outcome and survivorship of these patients were compared with those who received traditional exploratory laparotomic surgery. However, the laparoscopic group had identical PFS based on our limited cases compared with the laparotomic group. Although shorter OS can be observed in the laparoscopic group, significance was not evident. The same result was observed in the survivorship analysis. This means laparoscopic diagnosis, enucleation of OCCC, and surgical spillage did not worsen the outcome in these patients if direct laparotomic conversion with standard staging was followed by adjuvant chemotherapy.

Washing cytology obtained during surgery played a critical role in the outcome of OCCC based on the literature [12]. Because of the long study period, much of the data were missing, and only limited results for washing cytology were obtained. Similarly, a higher recurrent trend of malignant washing cytology was confirmed after surgery. According to the survival analysis, PFS was significantly worse in the group with malignant cytology. However, OS difference cannot be found in the same condition. Hence, malignant cytology may worsen only PFS and not OS.

This study had many limitations. This was a retrospective, single-institute, chart review study; therefore, the evidence obtained was not sufficient and comparable with that obtained through a prospective or multicenter retrospective study, such as Taiwanese Gynecological Oncology Group (TGOG) studies. The nonpaclitaxel-based group had fewer patients than the paclitaxel-based group because of the high compliance for paclitaxel therapy as per the current treatment guidelines of NCCN or ESMO. Only few cytology results could be obtained because of missing old records. However, based on our tentative and notable results, in the future, we would like to conduct a multicenter, retrospective study in Taiwan with the cooperation of TGOG by including a higher number of cases in order to obtain more reliable and practical results.

## 5. Conclusions

In conclusion, the necessity of adjuvant chemotherapy in stage IC OCCC is certain; however, platinum-based chemotherapy with or without paclitaxel in such cases demonstrated identical outcome and survivorship results. In cases where paclitaxel is unavailable (e.g., economic limitations and paclitaxel allergy), other alkylating agents (e.g., cyclophosphamide) can be considered as safe alternatives. There was no evidence for a worse outcome if the nonpaclitaxel regimen was chosen. Positive cytology indicates the requirement of more follow-ups because higher recurrence and shorter PFS are encountered in the daily practice in patients with OCCC.

## Figures and Tables

**Table 1:** Basic characteristics of the study groups.

**Factors**	**Chemotherapeutic Regimens**		
	**Paclitaxel-Based ^1^ (*n* = 64)**	**Nonpaclitaxel-Based ^2^ (*n* = 24)**	*p* *
Age	49.00 ± 8.419	48.50 ± 6.909	0.970
BMI (kg/m^2^)	22.35 ± 5.030	22.20 ± 2.719	0.472
LNs (number)	23.00 ± 13.04	23.50 ± 12.19	0.992
PFS (months)	47.5 ± 41.36	54.0 ± 53.9	0.157
OS (months)	53.5 ± 38.14	79.0 ± 49.42	0.070
	**Surgical Approach Methods**		
	**Laparotomy Staging (*n* = 76)**	**Laparoscopy Then Convert to Lapa Staging (*n* = 12)**	*p* *
Age	49.00 ± 7.69	47.00 ± 9.765	0.265
BMI (kg/m^2^)	22.35 ± 4.876	21.8 ± 2.66	0.701
LNs (number)	23.5 ± 13.19	22.5 ± 10.46	0.932
PFS (months)	49.5 ± 46.84	49.0 ± 35.55	0.719
OS (months)	56.5 ± 43.4	51.0 ± 32.77	0.600

BMI: body mass index (kg/m^2^), LNs: numbers of lymph nodes retrieved, PFS: progression-free survival, OS: overall survival, * *p* value: Student’s *t*-test or Wilcoxon–Mann–Whitney test. ^1^ Paclitaxel-based chemotherapy: paclitaxel (175 mg/m^2^) plus carboplatin (AUC 5–6). ^2^ Nonpaclitaxel-based chemotherapy: cisplatin (75 mg/m^2^) plus cyclophosphamide 750 (mg/m^2^) or carboplatin (AUC5) plus cyclophosphamide (600 mg/m^2^).

**Table 2:** Survivorship analysis between chemotherapies.

Outcomes	Chemotherapeutic Regimens		
	Paclitaxel-Based ^1^ (*n* = 64)	Nonpaclitaxel-Based ^2^ (*n* = 24)	*p* *
Recurrence			0.783
Yes	17	5	
No	47	19	
Survivorship			0.931
NED	52	20	
AWD	4	1	
DOD	8	3	

NED: no evidence of disease, AWD: alive with disease, DOD: dead of disease, * *p* value: chi-square test. ^1^ Paclitaxel-based chemotherapy: paclitaxel (175 mg/m^2^) plus carboplatin (AUC 5–6). ^2^ Nonpaclitaxel-based chemotherapy: cisplatin (75 mg/m^2^) plus cyclophosphamide 750 (mg/m^2^) or carboplatin (AUC5) plus cyclophosphamide (600 mg/m^2^).

**Table 3:** Survivorship analysis between surgical approaches.

Outcomes	Surgical Approaching Methods		
	Laparotomy Staging (*n* = 76)	Laparoscopy then Convert to Lapa Staging (*n* = 12)	*p* *
Recurrence			0.722
Yes	20	2	
No	56	10	
Survivorship			0.832
NED	62	10	
AWD	4	1	
DOD	10	3	

NED: no evidence of disease, AWD: alive with disease, DOD: dead of disease, * *p* value: chi-square test.

**Table 4:** Survivorship analysis between cytology results.

Outcomes	Washing Cytology Result		
	Positive (*n* = 13)	Negative (*n* = 38)	*p* *
Recurrence			0.068
Yes	6	7	
No	7	31	
Survivorship			0.157
NED	9	34	
AWD	2	1	
DOD	2	3	

NED: no evidence of disease, AWD: alive with disease, DOD: dead of disease, * *p* value: chi-square test.
